# Effects of soybean intercropping density on photosynthetic characteristics and disease resistance in tobacco

**DOI:** 10.3389/fpls.2025.1724956

**Published:** 2025-12-03

**Authors:** Xianglu Liu, Yanxia Hu, Chengwei Yang, Juan Li, Chunzhi Lu, Nengfei Tian, Haiyang Zhou, Shuangzhen Jin, Jiaen Su, Dexun Wang, Changhui Xu, Yukai Huang, Ming Liu

**Affiliations:** 1 Yunnan Tobacco Company Dali State Branch, Dali, Yunnan, China; 2 College of Agronomy and Biotechnology, Southwest University/Engineering Research Center of South Upland Agriculture, Ministry of Education, Chongqing, China

**Keywords:** tobacco–soybean intercropping, intercropping density, photosynthetic efficiency, carbon and nitrogen metabolism, disease resistance, flue-cured tobacco quality

## Abstract

**Background:**

Intercropping tobacco with soybean is a sustainable approach to improve resource use efficiency and crop resilience. However, the optimal soybean planting density for maximizing the physiological and protective benefits to tobacco remains unclear.

**Methods:**

A field experiment was conducted in Yunnan Province, China, including five treatments: tobacco monoculture and four tobacco–soybean intercropping densities. Photosynthetic parameters, carbon and nitrogen metabolism, defense-related physiology, and leaf chemical composition were measured across key growth stages.

**Results:**

Intercropping density significantly affected photosynthetic and metabolic performance in both species. The medium density with four soybean holes achieved the best results, increasing the net photosynthetic rate of tobacco by 30.8% compared with monoculture during the vigorous growth stage. This treatment also enhanced PSII photochemical efficiency, with Fv/Fm and ΦPSII values both higher than other treatments, and chlorophyll a content increased by 32.9% compared with high-density intercropping. The activities of Rubisco and nitrate reductase rose by 18.8% and 49.2%, respectively. At the same time, this density reduced the incidence of tobacco black shank disease and increased salicylic acid and jasmonic acid contents by 38.9% and 33.7%. Peroxidase, superoxide dismutase, and phenylalanine ammonia-lyase activities were also elevated. Tobacco leaves under this treatment showed a balanced chemical composition with high sugar, high potassium, and low chlorine contents, resulting in superior flue-cured quality and the highest economic return.

**Conclusion:**

The four-hole soybean density optimized photosynthesis, nitrogen metabolism, and defense responses, improving tobacco quality and yield. These findings provide a physiological and agronomic basis for developing efficient and sustainable tobacco–soybean intercropping systems.

## 1 Introduction

Tobacco (*Nicotiana tabacum* L.) is one of the most important economic crops in China, and its yield and quality are closely related to the sustainable development of the tobacco industry (Zhu et al., 2024). However, long-term monocropping has led to soil nutrient imbalance, frequent soil-borne diseases, and a decline in leaf quality, all of which have restricted the high-quality development of the tobacco sector (Wang et al., 2024). In recent years, intercropping and relay cropping systems have gained widespread attention as green and efficient cultivation practices, owing to their ability to improve soil conditions, enhance crop stress resistance, and increase overall productivity (Akchaya et al., 2025). Within tobacco production systems, how to enhance photosynthetic efficiency and optimize yield and quality through rational intercropping design has become a major research focus.

Soybean (*Glycine max* L.) - a typical leguminous crop - possesses the unique advantage of biological nitrogen fixation and can improve nitrogen availability in the soil (Kebede, 2021).Moreover, the differences in growth duration and ecological niches between soybean and tobacco promote a more efficient utilization of light and nutrient resources, and help to alleviate the obstacles caused by continuous tobacco cropping (Zhang et al., 2025). Recent studies have shown that tobacco-legume intercropping can improve soil fertility, promote nutrient accumulation in tobacco, and enhance economic returns. Tobacco - soybean intercropping significantly increased soil nutrient contents, with total nitrogen, available phosphorus, and available potassium all rising by more than 15% compared with monoculture (Gu et al., 2025). In addition, intercropping with soybean increased the populations of nitrifying and denitrifying bacteria in the tobacco rhizosphere, which promoted nitrogen cycling and improved the sugar-to-alkaloid and potassium-to-chlorine ratios in tobacco leaves, ultimately enhancing leaf quality (Tu, 2015).

Nevertheless, current research on tobacco-soybean intercropping has mainly focused on soil fertility improvement and nutrient interactions, whereas studies on the photosynthetic physiology and carbon–nitrogen metabolic regulation of tobacco under different intercropping conditions remain limited (Gu et al., 2025). The spatial configuration of an intercropping system directly influences canopy light distribution and energy balance. Inappropriate planting densities can intensify light competition, reduce light interception by tobacco, and suppress photosynthetic activity and carbon assimilation, ultimately disrupting the source–sink balance and lowering yield and quality (Wang et al., 2023). In contrast, a suitable intercropping density improves light capture and utilization, maintains the activity of photosynthetic organs, and promotes coordination between carbon and nitrogen metabolism, thereby enhancing the conversion and assimilation efficiency of photosynthetic products (Zhou et al., 2023a). Therefore, optimizing intercropping density is crucial for developing an efficient tobacco–soybean system. Understanding the photosynthetic responses of tobacco under different density configurations is essential for revealing interspecific light utilization mechanisms and improving resource-use efficiency and sustainability in tobacco production.

In addition, soil-borne diseases such as tobacco black shank frequently occur in continuous tobacco monocropping systems (Chen et al., 2023). Previous studies have indicated that intercropping can mitigate disease incidence by improving microclimate conditions and modifying rhizosphere microbial community structures (Boudreau, 2013; Yang et al., 2014). Moreover, intercropping can induce plant defense responses, including activation of the salicylic acid (SA) signaling pathway, enhancement of antioxidant enzyme activities, and stimulation of phenolic metabolism, thereby improving crop resistance (Barna et al., 2003; Gullner et al., 2017; Zheng et al., 2022b). However, the physiological and biochemical mechanisms underlying disease resistance regulation in tobacco under different intercropping densities remain poorly understood.

Yunnan Province is one of the major flue-cured tobacco-producing regions in China, and tobacco–soybean intercropping has already been adopted by local farmers (Gu et al., 2025). Nevertheless, due to the lack of systematic studies on intercropping density and spatial configuration, inappropriate soybean densities–either too high or too low–are commonly observed in production, leading to insufficient light interception by tobacco, decreased photosynthetic capacity, and reduced leaf quality. Previous studies have demonstrated that intercropping can enhance canopy photosynthetic efficiency and stress tolerance by optimizing light distribution, promoting carbon–nitrogen coordination, and activating defense-related physiological processes (Yao et al., 2017; Harman et al., 2021).

Therefore, in this study, the main flue-cured tobacco cultivar ‘Hongda’ commonly grown in Yunnan was used to establish a tobacco-soybean intercropping system with varying soybean densities. This study systematically analyzed the changes in photosynthetic physiology, carbon-nitrogen metabolism, and defense-related physiological traits of tobacco under different intercropping densities, aiming to elucidate the synergistic mechanisms of light energy utilization and disease resistance. The findings provide a theoretical basis for optimizing the design of tobacco-soybean intercropping systems, improving leaf quality, and promoting efficient resource utilization in Yunnan’s tobacco production.

## 2 Materials and methods

### 2.1 Experimental site

**Figure 1:**
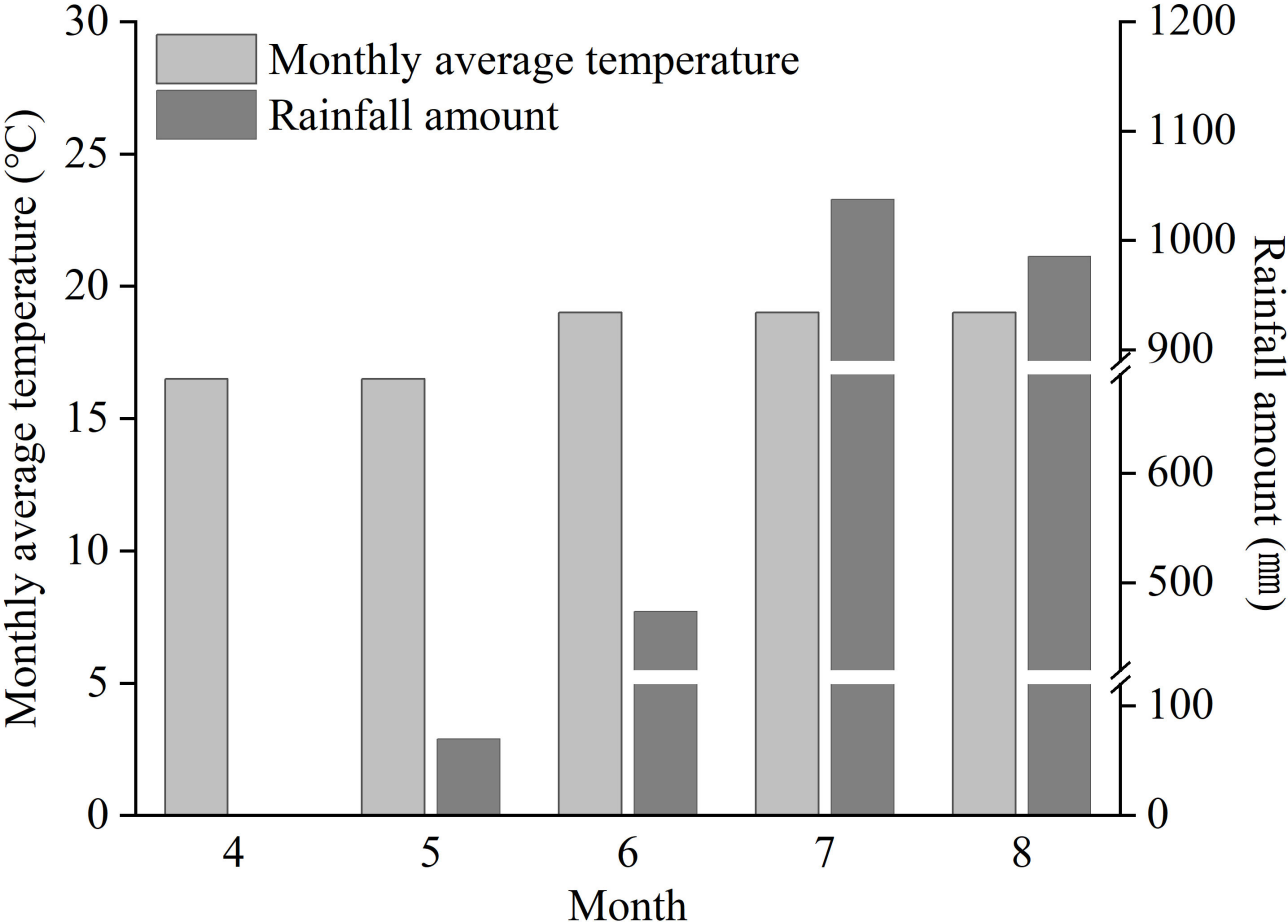
Temperature and rainfall during the tobacco growing period at the experimental site.

The field experiment was conducted from April to August 2025 in Midu County, Dali Bai Autonomous Prefecture, Yunnan Province, China (25.38°N, 100.41°E).The experimental area is characterized by a mid-subtropical monsoon climate, with an average annual temperature of 17.2 °C and an average annual precipitation of 642.5 mm. The monthly rainfall during the experimental period is shown in **Figure 1**, and the physicochemical properties of the experimental soil are presented in **Table 1**.

**Table 1:** Physicochemical properties of the soil at the experimental site.

TN (g/kg)	TP (g/kg)	TK (g/kg)	HN (mg/kg)	AP (mg/kg)	AK (mg/kg)
1.83 ± 0.07	1.23 ± 0.06	15.67 ± 0.91	198.91 ± 7.38	93.12 ± 4.07	717.12 ± 9.07

TN, total nitrogen; TP, total phosphorus; TK, total potassium; HN, hydrolyzable nitrogen; AP, available phosphorus; AK, available potassium. Values represent means ± standard deviations (n = 5).

### 2.2 Experimental design

The field experiment was conducted on flat land with relatively uniform soil fertility using a randomized complete block design (RCBD) with five treatments: tobacco monoculture (S0) and four tobacco–soybean intercropping densities (S2, S4, S6, and S8). Soybean was sown 15 days after tobacco transplanting, with two plants per hill. Intercropping density was adjusted by varying the number of soybean hills: two hills (S2, 6.6 × 10^4^ plants ha^-1^), four hills (S4, 1.32 × 10^5^ plants ha^-1^), six hills (S6, 1.98 × 10^5^ plants ha^-1^), and eight hills (S8, 2.64 × 10^5^ plants ha^-1^). Each treatment was replicated five times.

Tobacco seedlings were transplanted on April 26, 2025, and soybean was sown 15 days later. Soybean was planted on the ridge surface, 20 cm away from the tobacco stem base on both sides. In treatments S2, S4, S6, and S8, one, two, three, and four soybean hills were planted on each side of the tobacco plant, respectively. Each plot covered an area of 42.00 m², with a row spacing of 100 cm and a plant spacing of 50 cm. A 1 m-wide buffer zone was set between adjacent plots.

The experimental field had been continuously cultivated with flue-cured tobacco for three consecutive years prior to this study. After tobacco harvest in August of the previous year, no other crops were planted. Before transplanting, the soil was tilled and ridged following standard local tobacco field management practices. Other agronomic measures, including irrigation, fertilization, and pest control, were conducted in accordance with local production guidelines to ensure uniform management across all treatments and replicates. The main flue-cured tobacco cultivar ‘Hongda’ and the local soybean cultivar ‘Yunhuang No. 13’ were used in this study. These cultivars are widely planted in the Dali tobacco-growing region and represent typical varieties for local production systems.

### 2.3 Photosynthetic characteristics

Photosynthetic parameters of both tobacco and soybean were measured simultaneously at corresponding growth stages: the rosette stage (RS) of tobacco and the flowering stage (R2) of soybean; the fast-growing stage (FGS) of tobacco and the pod-filling stage (R4) of soybean; and the maturity stage (MS) of tobacco and the grain-filling stage (R6) of soybean. Measurements were conducted on clear days between 09:00 and 11:30 a.m. using a portable photosynthesis system (LI-6400XT, LI-COR, USA). The net photosynthetic rate (Pn), stomatal conductance (Gs), intercellular CO_2_ concentration (Ci), and transpiration rate (Tr) were determined on the third fully expanded leaf from the top of tobacco and the uppermost fully expanded trifoliolate leaf of soybean.

During measurement, the photosynthetic photon flux density (PPFD) was set at 1200 μmol·m^-2^·s^-1^. The reference CO_2_ concentration in the leaf chamber was maintained at 400 μmol·mol^-1^ using a CO_2_ cylinder, and the airflow rate was fixed at 500 μmol·s^-1^. The leaf chamber temperature was set at 25 °C, and relative humidity was maintained between 60% and 70% (Ding et al., 2025). Prior to measurement, leaves were illuminated for approximately 10 minutes to reach a steady photosynthetic state. Each leaf was measured at three different positions, and the average value was calculated. Five plants were measured per treatment.

On the same day as photosynthetic measurements, leaves were dark-adapted for 30 minutes, and the maximal photochemical efficiency of photosystem II (Fv/Fm) was determined using a JUNIOR-PAM chlorophyll fluorometer (Heinz Walz, Germany). Under light-adapted conditions (identical to those used for photosynthetic measurements), the actual photochemical quantum yield (ΦPSII) was recorded.

For pigment analysis, leaf discs were sampled from the same leaves used for photosynthetic gas exchange measurements. Pigments were extracted with 80% acetone, and the concentrations of chlorophyll a, chlorophyll b, and carotenoids were determined spectrophotometrically according to the method of Lichtenthaler and Wellburn (1983).

### 2.4 Activities of key enzymes involved in carbon and nitrogen metabolism

Leaves previously used for photosynthetic measurements were sampled to determine the activities of key enzymes involved in carbon and nitrogen metabolism. Ribulose-1,5-bisphosphate carboxylase/oxygenase (Rubisco) activity was measured using the NADH-coupled spectrophotometric method at 340 nm (Li et al., 2025). Nitrate reductase (NR) activity was determined by the Griess colorimetric method at 540 nm (Imran et al., 2019). Glutamine synthetase (GS) activity was assayed using the hydroxylamine colorimetric method at 540 nm (Peng et al., 2016).

### 2.5 Disease assessment

The incidence of tobacco black shank disease (*Phytophthora nicotianae*) was investigated every 15 days starting 30 days after transplanting and continued until 90 days. In each plot, disease occurrence was assessed using the five-point sampling method on 20 randomly selected plants. Disease severity was recorded according to the *Grading and Investigation Method for Tobacco Diseases and Insect Pests* (GB/T 23222–2008). The disease incidence and disease index were calculated as follows:


Disease incidence (%)=[Number of diseased plants/Total number of surveyed plants]×100;

Disease index (%)=[∑(Disease severity grade×Number of plants at that grade)/(Maximum disease grade×Total number of surveyed plants)]×100

Based on the disease index obtained from five consecutive surveys, the area under the disease progress curve (AUDPC) was calculated using the following formula:


$$\begin{array}{c} AUDPC=\sum_{i=1}^{n-1}[\frac{\left(DI_{i+1}+DI_{i}\right)}{2}\times (t_{i+1\;}\;-\;t_{i})] \end{array}$$

*i* = the order of the survey (from the first to the (n-1)th observation); $DI_{i+1}+DI_{i}$ = the disease index recorded at the *i* and i+1  surveys, respectively; $t_{i}$ and $t_{i+1\;}$ = the number of days after transplanting (DAT) at the *i* and i+1  surveys; *n* = the total number of surveys.

### 2.6 Determination of defense-related physiological parameters

Defense-related physiological parameters of tobacco were measured during the fast-growing stage. The concentrations of salicylic acid (SA) and jasmonic acid (JA) were quantified using enzyme-linked immunosorbent assay (ELISA) kits (Shanghai Youxuan Biotechnology Co., Ltd., China) following the manufacturer’s protocol. Peroxidase (POD) activity was determined by the guaiacol oxidation method (Li and Gong, 2008). Superoxide dismutase (SOD) activity was assayed using the nitroblue tetrazolium (NBT) photochemical reduction method (Qu et al., 2014). Phenylalanine ammonia-lyase (PAL) activity was measured spectrophotometrically at 290 nm using L-phenylalanine as the substrate, and expressed as the rate of trans-cinnamic acid formation (Jin et al., 2009). Total polyphenol (TP) content was determined by the Folin–Ciocalteu colorimetric method (Cheng et al., 2022).

### 2.7 Tobacco leaf quality and yield assessment

After flue-curing, 2 kg of middle tobacco leaves (C3F grade) were collected from each treatment to determine the chemical composition. Total and reducing sugar contents were measured according to YC/T 159–2002 (State Tobacco Monopoly Administration, 2002a), total nitrogen according to YC/T 161–2002 (State Tobacco Monopoly Administration, 2002b), total alkaloids according to YC/T 468–2013 (State Tobacco Monopoly Administration, 2013), potassium content according to YC/T 217–2007 (State Tobacco Monopoly Administration, 2007), and chlorine content according to YC/T 162–2011 (State Tobacco Monopoly Administration, 2011).

In early September 2025, the flue-cured tobacco leaves from each treatment were graded according to GB 2635–1992. The yield, output value, proportion of high- and middle-grade leaves, and average price were recorded. Tobacco prices were based on the local purchase prices set by the Tobacco Company, while soybean income was calculated according to market purchase prices and measured yields.

### 2.8 Data processing and statistical analysis

Experimental data were initially organized and calculated using Microsoft Excel 2019. Statistical analyses were performed using SPSS 26.0 software. Two-way analysis of variance (ANOVA) was applied to evaluate the effects of intercropping density, growth stage, and their interactions. For data involving a single factor, one-way ANOVA was used. Significant differences among treatments were determined by Duncan’s multiple range test at *P* < 0.05. All figures and visualizations were prepared using Origin 2022 software.

## 3 Results

### 3.1 Photosynthetic parameters of tobacco and soybean

**Figure 2:**
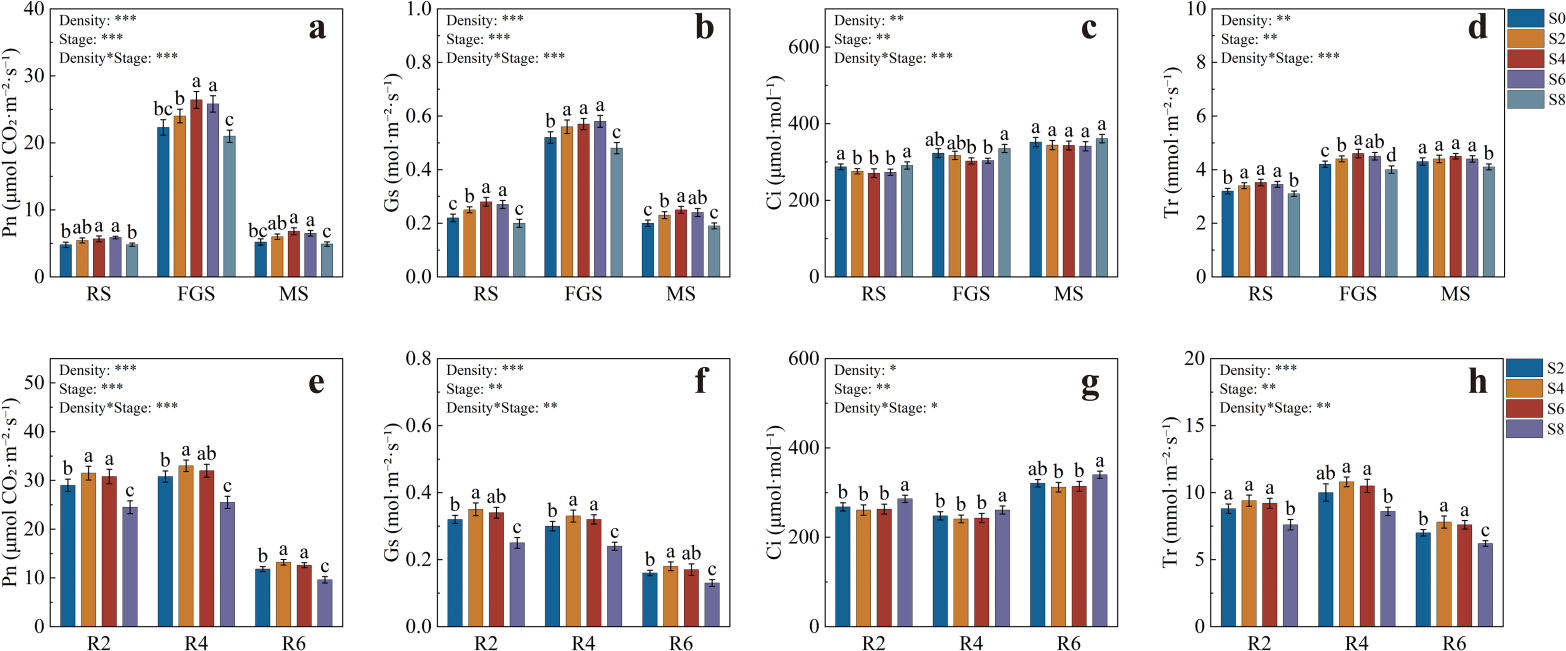
Photosynthetic parameters of tobacco and soybean under different intercropping densities. **(a)** net photosynthetic rate of tobacco; **(b)** stomatal conductance of tobacco; **(c)** intercellular CO_2_ concentration of tobacco; **(d)** transpiration rate of tobacco; **(e)** net photosynthetic rate of soybean; **(f)** stomatal conductance of soybean; **(g)** intercellular CO_2_ concentration of soybean; **(h)** transpiration rate of soybean. RS, rosette stage of tobacco; FGS, fast-growing stage of tobacco; MS, maturity stage of tobacco. Different lowercase letters indicate significant differences at *P* < 0.05. *, **, and *** represent significance at *P* < 0.05, *P* < 0.01, and *P* < 0.001, respectively.

The gas exchange parameters of tobacco are presented in **Figures 2a–d**. During the RS stage, Pn values were generally low across treatments, but S4 and S6 were significantly higher than S0 and S8 (**Figure 2a**). At the FGS stage, Pn increased markedly and reached its maximum across the growth period, with S4 and S6 attaining 26.41 and 25.80 μmol CO_2_·m^-2^·s^-1^, respectively, both significantly higher than the other treatments. At the MS stage, Pn decreased overall, yet S4 and S6 maintained higher values, being 30.77% and 25.00% greater than S0, respectively. The variation of Gs followed a pattern similar to Pn (**Figure 2b**). During the RS stage, S2, S4, and S6 were significantly higher than S0 and S8. At the FGS stage, Gs further increased, reaching 0.56, 0.57, and 0.58 mol·m^-2^·s^-1^ in S2, S4, and S6, respectively, all significantly higher than S0 and S8. During the MS stage, Gs declined, but S4 remained significantly higher than S0, S2, and S8. Ci at the RS stage was 287.5 and 291 μmol·mol^-1^ for S0 and S8, respectively, both significantly higher than S2, S4, and S6 (**Figure 2c**). At the FGS stage, Ci in S8 rose to 335.2 μmol·mol^-1^, significantly higher than in S4 and S6, while no significant differences were found among treatments at the MS stage. Tr at the RS stage was significantly higher in S2, S4, and S6 than in S0 and S8 (**Figure 2d**). During the FGS stage, Tr increased, with S4 showing the highest value of 4.60 mmol·m^-2^·s^-1^, 9.52% higher than S0, while S8 showed the lowest value of 4.01 mmol·m^-2^·s^-1^. At the MS stage, Tr in S8 remained significantly lower than the other treatments.

The gas exchange parameters of soybean are shown in **Figures 2e–h**. At the R2 stage, Pn was highest in S4 and S6, reaching 31.51 and 30.82 μmol CO_2_·m^-2^·s^-1^, respectively, both significantly higher than S2 and S8 (**Figure 2e**). At the R4 stage, Pn increased slightly, with S4 reaching 33.04 μmol CO_2_·m^-2^·s^-1^, significantly higher than S2 and S8. At the R6 stage, Pn decreased overall, but S4 and S6 remained significantly higher than S8 by 37.5% and 31.25%, respectively. Gs at the R2 stage was highest in S4, reaching 0.35 mol·m^-2^·s^-1^, significantly higher than S2 and S8 (**Figure 2f**), while S6 did not differ significantly from S4. At the R4 stage, Gs in S4 and S6 remained significantly higher than in S2 and S8, and though Gs declined at the R6 stage, it remained highest in S4. For Ci, S8 exhibited the highest value of 286.3 μmol·mol^-1^ at the R2 stage, significantly higher than other treatments (**Figure 2g**). Ci in S4 and S6 was lower, at 261.3 and 268.4 μmol·mol^-1^, respectively, showing the same pattern at the R4 stage, with S8 remaining significantly highest. At the R6 stage, Ci in S8 increased further to 335.2 μmol·mol^-1^, significantly higher than in S4 and S6. At the R2 stage, Tr was highest in S4 and S6, reaching 9.40 and 9.21 mmol·m^-2^·s^-1^, respectively, both significantly higher than S2 and S8 (**Figure 2h**). At the R4 stage, Tr further increased, with S4 and S6 being 25.81% and 22.58% higher than S8, respectively. During the R6 stage, Tr decreased, but S4 and S6 still maintained significantly higher values than S2 and S8.

In summary, S4 and S6 consistently maintained higher Pn, Gs, and Tr values, along with lower Ci, throughout the growth period of both tobacco and soybean. This indicates that appropriate intercropping density effectively enhanced photosynthetic carbon assimilation and gas exchange coordination in the system.

### 3.2 Chlorophyll fluorescence parameters of tobacco and soybean

Different intercropping densities, growth stages, and their interactions had significant effects on the chlorophyll fluorescence parameters of both tobacco and soybean (**Tables 2**, **3**). The chlorophyll fluorescence parameters of tobacco are presented in **Table 2**. During the RS stage, Fv/Fm values ranged from 0.77 to 0.80, with S4 significantly higher than S0 and S8. After entering the FGS stage, Fv/Fm slightly decreased across treatments, with S4 at 0.74, still significantly higher than the others. By the MS stage, Fv/Fm declined further, but S4 maintained the highest value at 0.69, representing an 11.29% increase compared with S8. ΦPSII showed that S4 and S6 were significantly higher than S0, S2, and S8 during all three stages (RS, FGS, and MS). Across all stages, ΦPSII first increased and then decreased, reaching its maximum at the FGS stage, where S4 and S6 were 19.44% and 16.67% higher than S0, respectively. The chlorophyll fluorescence parameters of soybean are shown in **Table 3**. At the R2 and R4 stages, Fv/Fm in S4 was significantly higher than in S2 and S8, and at the R6 stage, S4 still maintained the highest value, significantly exceeding S8. ΦPSII in S4 was 7.69% higher than in S8 at the R2 stage and increased slightly at the R4 stage. During the R4 and R6 stages, S4 remained significantly higher than S2 and S8. Overall, S4 and S6 treatments consistently maintained higher Fv/Fm and ΦPSII values throughout the growth period of both tobacco and soybean, indicating enhanced photochemical efficiency and more stable PSII function under moderate intercropping density.

**Table 2:** Chlorophyll fluorescence parameters of tobacco under different soybean intercropping densities.

Stage	Treatments	Fv/Fm	ΦPSII
RS	S0	0.78 ± 0.01^b^	0.34 ± 0.02^b^
	S2	0.79 ± 0.01^ab^	0.34 ± 0.02^b^
	S4	0.80 ± 0.01^a^	0.36 ± 0.02^a^
	S6	0.79 ± 0.01^ab^	0.37 ± 0.02^a^
	S8	0.77 ± 0.01^b^	0.33 ± 0.02^b^
FGS	S0	0.71 ± 0.01^bc^	0.36 ± 0.02^c^
	S2	0.73 ± 0.01^ab^	0.39 ± 0.01^b^
	S4	0.74 ± 0.02^a^	0.43 ± 0.02^a^
	S6	0.72 ± 0.02^bc^	0.42 ± 0.02^a^
	S8	0.68 ± 0.01^c^	0.34 ± 0.02^d^
MS	S0	0.65 ± 0.01^bc^	0.20 ± 0.01^bc^
	S2	0.67 ± 0.01^ab^	0.21 ± 0.01^b^
	S4	0.69 ± 0.01^a^	0.23 ± 0.01^a^
	S6	0.68 ± 0.02^ab^	0.23 ± 0.01^a^
	S8	0.62 ± 0.01^c^	0.18 ± 0.01^c^
Density		**	**
Stage		*	**
Density*Stage		**	***

Data are presented as mean ± standard deviation (SD). *, **, and *** indicate significant differences at *P* < 0.05, *P* < 0.01, and *P* < 0.001, respectively. Different lowercase letters within the same column indicate significant differences at *P* < 0.05 (the same below).

**Table 3:** Chlorophyll fluorescence parameters of soybean under different soybean intercropping densities.

Stage	Treatments	Fv/Fm	ΦPSII
R2	S2	0.76 ± 0.01^b^	0.41 ± 0.01^b^
	S4	0.77 ± 0.01^a^	0.42 ± 0.01^a^
	S6	0.76 ± 0.01^ab^	0.41 ± 0.01^b^
	S8	0.74 ± 0.01^c^	0.39 ± 0.02^c^
R4	S2	0.79 ± 0.01^b^	0.44 ± 0.01^b^
	S4	0.81 ± 0.01^a^	0.45 ± 0.01^a^
	S6	0.81 ± 0.01^ab^	0.45 ± 0.02^ab^
	S8	0.79 ± 0.01^b^	0.42 ± 0.02^c^
R6	S2	0.78 ± 0.01^ab^	0.44 ± 0.01^b^
	S4	0.79 ± 0.01^a^	0.45 ± 0.01^a^
	S6	0.78 ± 0.01^ab^	0.45 ± 0.01^a^
	S8	0.77 ± 0.01^b^	0.42 ± 0.02^c^
Density		**	**
Stage		*	*
Density*Stage		*	**

Note: Data are presented as mean ± standard deviation (SD). *, **, and *** indicate significant differences at P < 0.05, P < 0.01, and P < 0.001, respectively. Different lowercase letters within the same column indicate significant differences at P < 0.05 (the same below).

### 3.3 Photosynthetic pigment contents of tobacco and soybean

**Figure 3:**
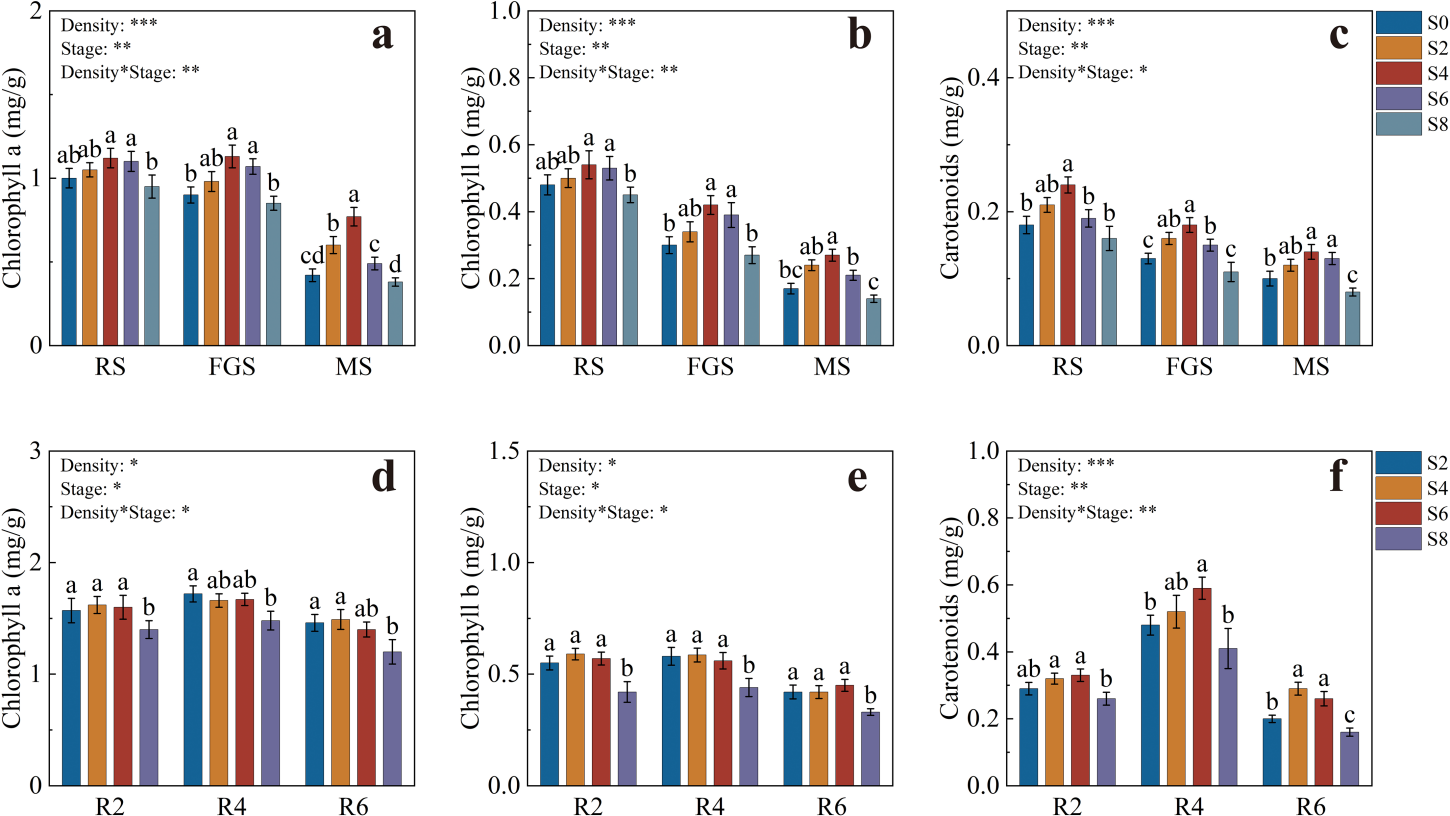
Pigment contents of tobacco and soybean at different growth stages under various intercropping densities. **(a)** chlorophyll a content of tobacco; **(b)** chlorophyll b content of tobacco; **(c)** carotenoid content of tobacco; **(d)** chlorophyll a content of soybean; **(e)** chlorophyll b content of soybean; **(f)** carotenoid content of soybean. Different lowercase letters indicate significant differences at *P* < 0.05. *, **, and *** represent significance at *P* < 0.05, *P* < 0.01, and *P* < 0.001, respectively.

The pigment contents of tobacco leaves are shown in **Figures 3a–c**. Chlorophyll a levels were generally higher during the RS and FGS stages, with S4 and S6 significantly exceeding S8. At the FGS stage, chlorophyll a in S4 was 32.94% higher than in S8. By the MS stage, pigment contents decreased across all treatments, but chlorophyll a in S4 remained 1.83 and 2.06 times higher than in S0 and S8, respectively (**Figure 3a**). Chlorophyll b showed a similar trend to chlorophyll a during the RS and FGS stages, with S4 and S6 significantly higher than S0 and S8. At the FGS stage, chlorophyll b in S4 was 55.56% higher than in S8, followed by a pronounced decline at the MS stage (**Figure 3b**). Carotenoid content was highest in S4 during the RS stage (0.24 mg·g^-1^), significantly higher than S0, S6, and S8. Although carotenoid content declined during the FGS and MS stages, S4 consistently remained the highest among treatments (**Figure 3c**).

The pigment contents of soybean leaves are presented in **Figures 3d–f**. At the R2 stage, chlorophyll a in S2, S4, and S6 was significantly higher than in S8, increasing by 12.14%, 15.71%, and 14.29%, respectively. At the R4 stage, only S2 was significantly higher than S8. By the R6 stage, S2 and S4 reached the highest levels, at 1.46 and 1.49 mg·g^-1^, respectively (**Figure 3d**). Chlorophyll b showed a similar trend across the R2, R4, and R6 stages, with S2, S4, and S6 significantly higher than S8. At the R2 stage, these treatments were 30.95%, 40.48%, and 35.71% higher than S8, respectively (**Figure 3e**). Carotenoid content in S4 and S6 reached 0.32 and 0.33 mg·g^-1^ at the R2 stage, both significantly higher than S8. During the R4 stage, carotenoid levels increased significantly across all treatments, with S6 reaching the highest value of 0.59 mg·g^-1^. At the R6 stage, carotenoid content declined markedly, but S4 and S6 still remained significantly higher than S2 and S8 (**Figure 3f**).

Overall, S4 and S6 treatments maintained higher levels of chlorophylls and carotenoids throughout the growth period of both tobacco and soybean, providing a stable pigment foundation for sustained and efficient photosynthetic performance.

### 3.4 Activities of key enzymes involved in carbon and nitrogen metabolism in tobacco

**Figure 4:**
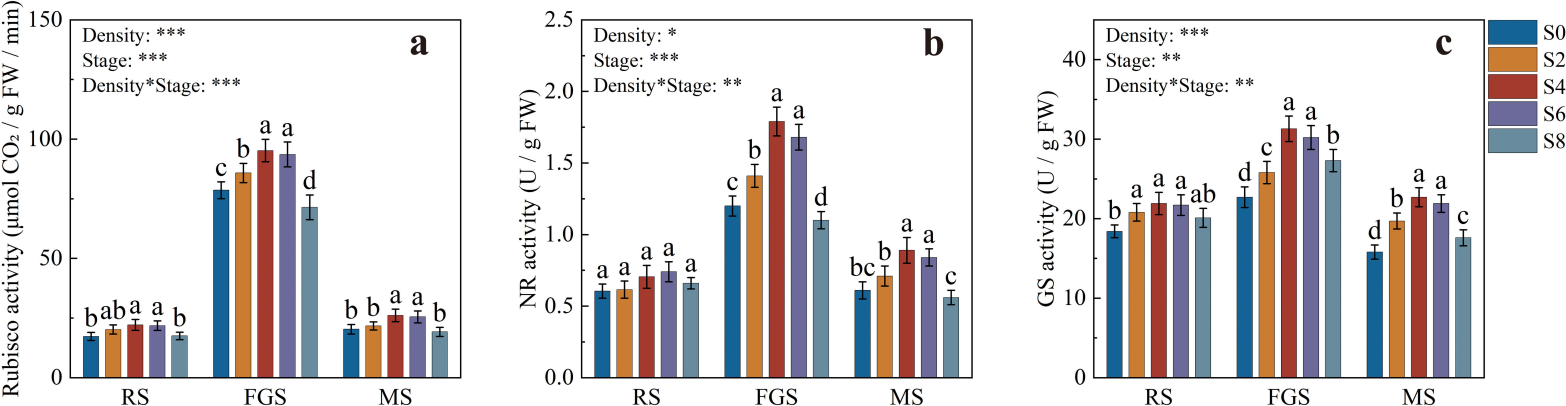
Activities of key enzymes involved in carbon and nitrogen metabolism in tobacco under different intercropping densities. **(a)** ribulose-1,5-bisphosphate carboxylase/oxygenase (Rubisco) activity; **(b)** nitrate reductase (NR) activity; **(c)** glutamine synthetase (GS) activity. Different lowercase letters indicate significant differences at *P* < 0.05. *, **, and *** represent significance at *P* < 0.05, *P* < 0.01, and *P* < 0.001, respectively.

The activities of Rubisco, nitrate reductase (NR), and glutamine synthetase (GS) were significantly influenced by intercropping density and growth stage (**Figures 4a–c**). Overall, the three enzymes exhibited a consistent trend of “promotion under moderate density and inhibition under excessive density.” During the RS stage, Rubisco activity in S4 and S6 was significantly higher than in S0 and S8. At the FGS stage, Rubisco activity reached its maximum, with S4 and S6 showing 18.75% and 16.25% increases over S0, respectively. By the MS stage, Rubisco activity declined in all treatments (**Figure 4a**). NR activity also varied significantly across growth stages, with S4 and S6 maintaining the highest levels during both the FGS and MS stages. At the FGS stage, NR activity in S4 was 49.17% higher than in S0 (**Figure 4b**). GS activity followed a similar pattern to Rubisco and NR, peaking at the FGS stage. S4 and S6 were significantly higher than S0, S2, and S8. Although GS activity decreased during the MS stage, S4 and S6 still maintained relatively higher levels (**Figure 4c**). Overall, S4 and S6 treatments substantially enhanced the activities of Rubisco, NR, and GS, thereby promoting carbon assimilation and nitrogen metabolism in tobacco under optimal intercropping density.

### 3.5 Investigation and evaluation of tobacco black shank disease

The incidence rate and disease index of tobacco black shank increased significantly with the number of days after transplanting, but clear differences were observed among intercropping densities (**Table 4**). Throughout the investigation period, S4 exhibited the lowest disease occurrence, with both incidence rate and disease index remaining significantly lower than those of other treatments. At 90 days after transplanting, the incidence rate and disease index of S4 were 19.0% and 27.3, respectively, representing reductions of 47.22% and 47.30% compared with monocropped tobacco. Disease severity in S2 and S6 was also significantly reduced, although still higher than in S4. In contrast, S8 showed much greater susceptibility to black shank infection.

**Table 4:** Incidence rate and disease index of tobacco black shank under different soybean intercropping densities.

Treatments	DAT 30		DAT 45		DAT 60		DAT 75		DAT 90	
	Disease incidence (%)	Disease index	Disease incidence (%)	Disease index	Disease incidence (%)	Disease index	Disease incidence (%)	Disease index	Disease incidence (%)	Disease index
S0	5.0 ± 0.7^b^	9.0 ± 5.9^b^	18.0 ± 1.1^b^	29.4 ± 7.6^b^	29.0 ± 0.8^b^	39.8 ± 5.7^b^	33.0 ± 0.6^b^	46.4 ± 3.0^a^	36.0 ± 0.8^a^	51.8 ± 4.6^b^
S2	1.0 ± 0.5^c^	1.8 ± 3.2^c^	10.0 ± 0.7^c^	18.4 ± 4.9^c^	18.0 ± 0.6^c^	26.3 ± 3.2^c^	26.0 ± 0.8^c^	34.1 ± 4.6^b^	28.0 ± 0.6^b^	38.7 ± 3.6^c^
S4	0.0 ± 0.0^d^	1.0 ± 1.5^c^	6.0 ± 0.5^d^	11.1 ± 2.7^d^	12.0 ± 0.6^d^	18.3 ± 2.5^d^	17.0 ± 0.6^e^	23.4 ± 2.6^c^	19.0 ± 0.5^c^	27.3 ± 1.8^d^
S6	1.0 ± 0.5^c^	3.4 ± 5.7^c^	7.0 ± 0.6^d^	14.4 ± 2.8^cd^	17.0 ± 0.6^c^	24.8 ± 3.0^c^	22.0 ± 0.6^d^	29.3 ± 2.7^b^	26.0 ± 0.5^b^	33.3 ± 3.1^cd^
S8	11.0 ± 0.8^a^	22.1 ± 6.1^a^	32.0 ± 1.1^a^	44.5 ± 6.7^a^	39.0 ± 0.5^a^	59.0 ± 4.0^a^	36.0 ± 0.8^a^	54.3 ± 4.2^a^	38.0 ± 0.6^a^	62.0 ± 2.3^a^

Different lowercase letters within the same column indicate significant differences at *P* < 0.05.

**Figure 5:**
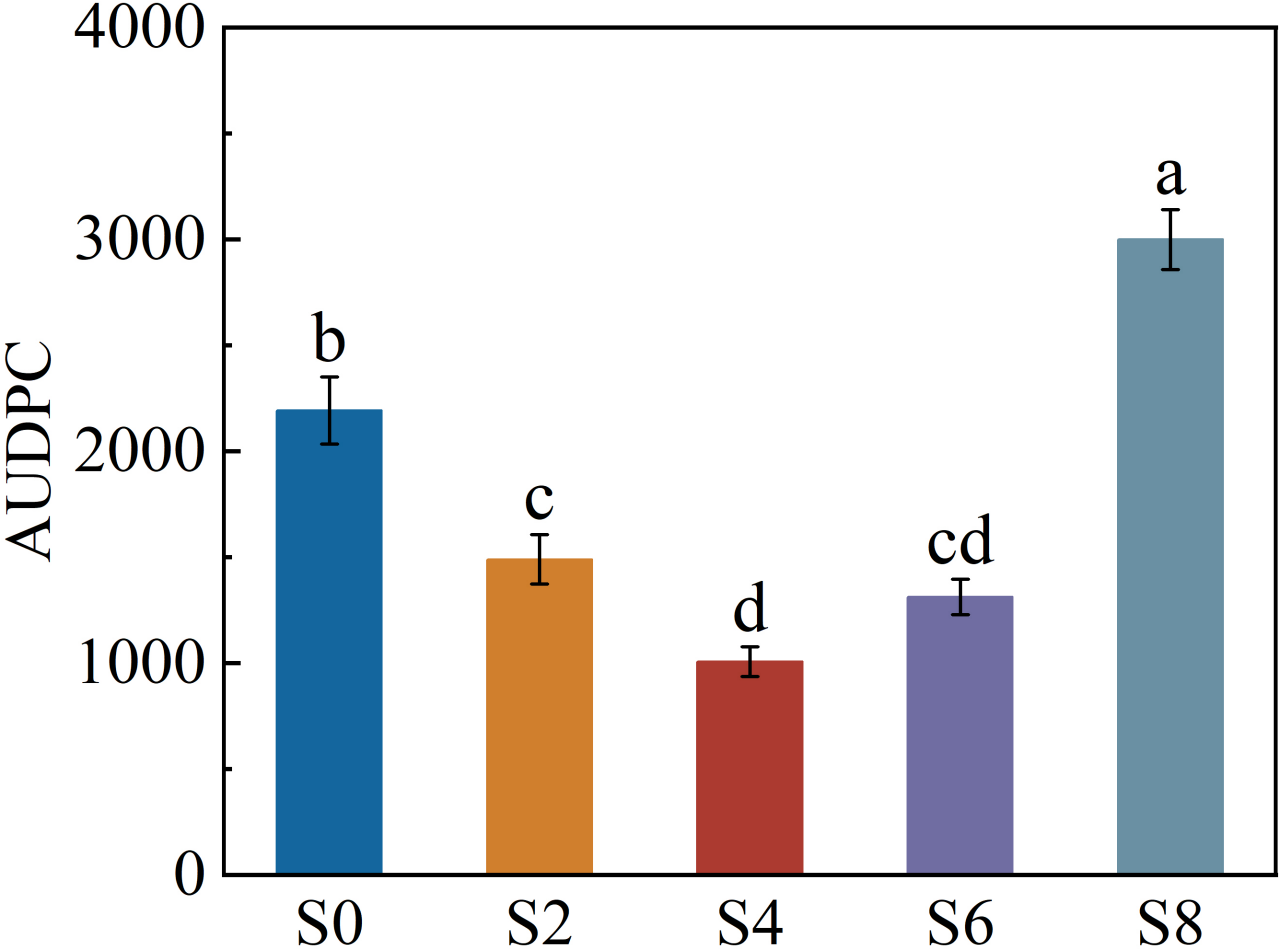
Area under the disease progress curve (AUDPC) for tobacco black shank under different intercropping densities. Different lowercase letters indicate significant differences at *P* < 0.05. *, **, and *** represent significance at *P* < 0.05, *P* < 0.01, and *P* < 0.001, respectively.

After integrating the disease index across the entire infection period, the area under the disease progress curve (AUDPC) differed significantly among treatments (**Figure 5**). The AUDPC value of S4 was the lowest, significantly smaller than that of all other treatments. No significant difference was found between S2 and S6, but both recorded markedly lower values than the monocropping treatment. In contrast, S8 exhibited the highest AUDPC value. Overall, S2, S4, and S6 effectively suppressed the incidence and progression of tobacco black shank disease, whereas S8 intensified disease development.

### 3.6 Defense-related enzyme activities and signal molecule contents in tobacco

**Figure 6:**
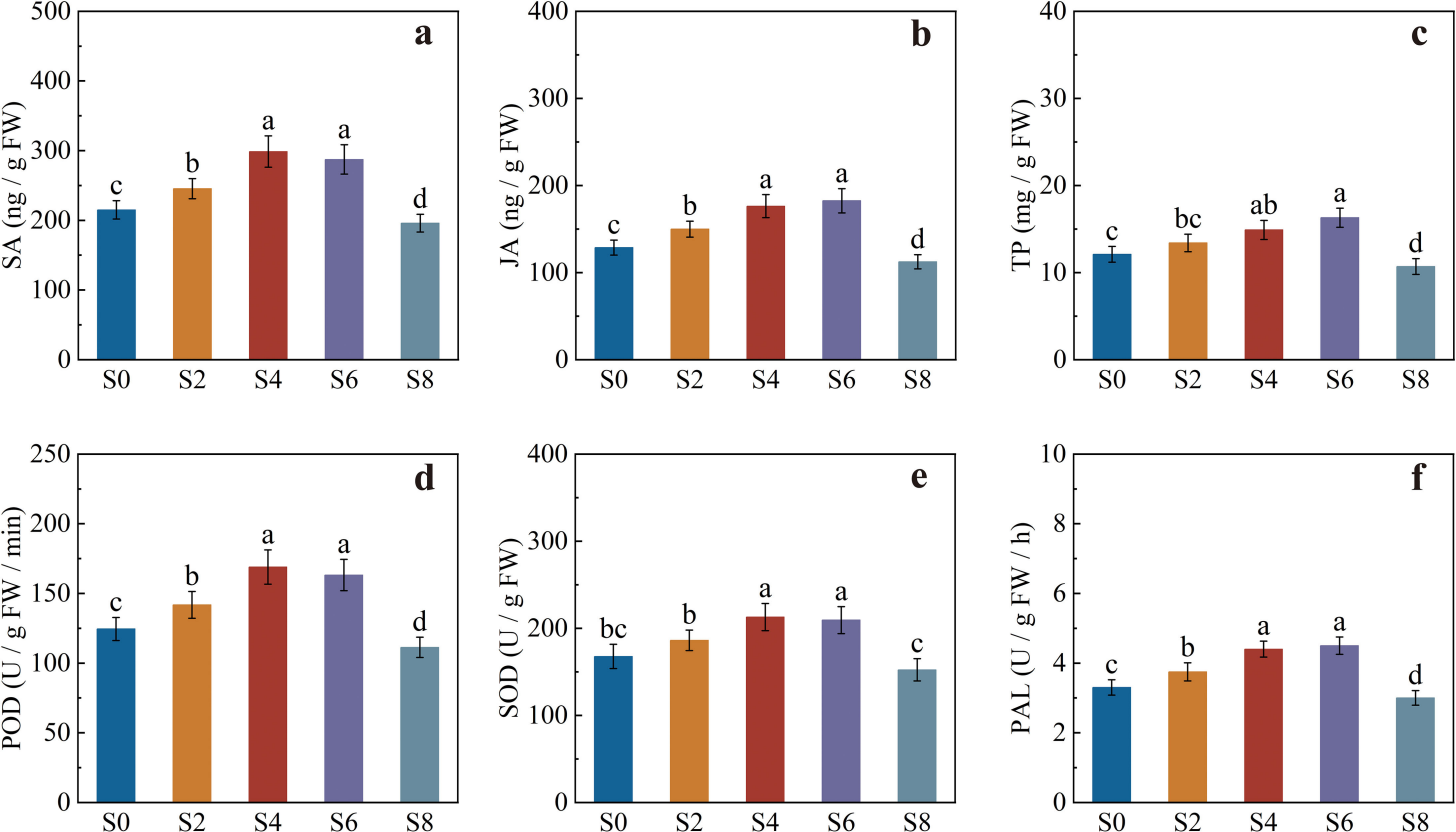
Defense-related enzyme activities and signal molecule contents in tobacco leaves under different intercropping densities. **(a)** salicylic acid (SA) content; **(b)** jasmonic acid (JA) content; **(c)** total polyphenol (TP) content; **(d)** peroxidase (POD) activity; **(e)** superoxide dismutase (SOD) activity; **(f)** phenylalanine ammonia-lyase (PAL) activity. Different lowercase letters indicate significant differences at *P* < 0.05.

Significant differences were observed among treatments in the defense-related physiological parameters (**Figure 6**). The contents of salicylic acid (SA) and jasmonic acid (JA) were significantly influenced by intercropping density, showing a trend of “enhancement under moderate density and reduction under excessive density” (**Figures 6a, b**). Compared with monocropping, S2, S4, and S6 markedly increased the SA and JA contents in leaves, with the most pronounced effects in S4 and S6. The SA contents in S4 and S6 were 38.93% and 33.67% higher than those in S0, respectively. However, SA and JA levels in S8 were significantly lower than in monocropping.

Total polyphenol (TP) content showed a pattern similar to that of SA and JA, exhibiting a significant increase under the S6 treatment, which was 21.43% higher than S0 (**Figure 6c**). Meanwhile, peroxidase (POD) and superoxide dismutase (SOD) activities were significantly higher in S4 and S6 than in S0, whereas POD activity in S8 was significantly lower than in S0 (**Figures 6d, e**). Phenylalanine ammonia-lyase (PAL) activity was also highest in S4 and S6, being 33.33% and 36.36% higher than in monocropping, respectively. S2 showed a moderate increase, while S8 exhibited a marked decline, consistent with the trend observed for TP content (**Figure 6f**).

In summary, S4 and S6 treatments substantially enhanced SA, JA, and TP contents as well as POD, SOD, and PAL activities in tobacco, thereby strengthening both systemic and non-specific defense responses. In contrast, excessive intercropping density (S8) suppressed these physiological processes, weakening the overall defense capacity of tobacco.

### 3.7 Chemical quality and economic benefits of flue-cured tobacco

Significant differences were observed in the chemical composition of flue-cured tobacco leaves among treatments (**Table 5**). For sugar content, variations in total and reducing sugars followed a similar pattern. S4 and S6 exhibited the highest total sugar contents, reaching 26.84% and 26.91%, respectively, both significantly higher than S0 and S2. The reducing sugar content in S4 reached 17.26%, significantly higher than in all other treatments, whereas S8 showed no significant difference compared with S0 and S2. Regarding nitrogenous compounds, total alkaloid content did not differ significantly among treatments. However, total nitrogen content was significantly higher in S4, S6, and S8 compared with S0. Potassium content was highest in S4 (3.02%), significantly higher than in S0 and S8, while chlorine content was greatest in S0 and S8 and lowest in S4. Overall, S4 displayed the most favorable balance among key chemical quality indicators, characterized by high sugar, high potassium, and moderate nitrogen–alkaloid levels, which correspond to superior flue-cured tobacco quality.

**Table 5:** Chemical quality of flue-cured tobacco leaves under different soybean intercropping densities.

Treatments	Total sugar (%)	Reducing sugar (%)	Total nitrogen (%)	Total alkaloids (%)	Potassium (%)	Chlorine (%)
S0	23.12 ± 1.32^c^	14.23 ± 0.98^b^	1.49 ± 0.06^b^	2.32 ± 0.11^a^	2.77 ± 0.11^b^	0.27 ± 0.02^a^
S2	25.35 ± 1.08^b^	15.12 ± 0.85^b^	1.52 ± 0.05^ab^	2.35 ± 0.10^a^	2.85 ± 0.12^ab^	0.25 ± 0.02^ab^
S4	26.84 ± 1.01^a^	17.26 ± 0.91^a^	1.56 ± 0.07^a^	2.38 ± 0.12^a^	3.02 ± 0.14^a^	0.22 ± 0.01^b^
S6	26.91 ± 1.35^a^	16.58 ± 0.88^ab^	1.58 ± 0.06^a^	2.45 ± 0.11^a^	2.83 ± 0.13^ab^	0.25 ± 0.02^ab^
S8	25.98 ± 1.40^ab^	14.95 ± 1.02^b^	1.59 ± 0.08^a^	2.42 ± 0.13^a^	2.71 ± 0.15^b^	0.28 ± 0.03^a^

Different lowercase letters within the same column indicate significant differences at *P* < 0.05.

Different intercropping densities had significant effects on the economic performance of the tobacco–soybean system (**Table 6**). In terms of tobacco yield, there were no significant differences among S0, S2, S4, and S6, whereas S8 showed a marked reduction. The proportion of high-grade leaves was highest in S0 and lowest in S8. The trend in tobacco output value followed a pattern similar to yield, with S0 and S4 significantly higher than S8. In contrast, soybean output value showed the opposite trend: S8 achieved the highest soybean income, significantly exceeding the other intercropping treatments, while S2 was the lowest. From the perspective of total system productivity, S4 and S6 achieved the highest overall economic returns, with total output values significantly higher than S0. Although S8 had the lowest tobacco output, its combined system value did not differ significantly from the other treatments. These results indicate that S4 and S6 provided the optimal balance between tobacco yield and soybean profitability, resulting in the greatest overall system benefit.

**Table 6:** Yield and economic benefits of the tobacco–soybean intercropping system under different soybean intercropping densities.

Treatments	Tobacco yield (kg/ha)	Proportion of high-grade leaves (%)	Tobacco output value (CNY/ha)	Soybean output value (CNY/ha)	Total system output value (CNY/ha)
S0	2490.52 ± 89.51^a^	67.51 ± 2.82^a^	87691.20 ± 1450.54^a^		87691.20 ± 1450.54^b^
S2	2480.81 ± 76.83^a^	59.59 ± 2.52^bc^	85216.16 ± 1786.58^ab^	5292.448 ± 237.13^d^	90508.61 ± 1803.12^ab^
S4	2505.24 ± 95.18^a^	64.22 ± 3.03^ab^	86881.72 ± 1550.88^a^	9293.928 ± 543.19^c^	96175.65 ± 1638.02^a^
S6	2456.51 ± 85.27^a^	60.83 ± 2.26^bc^	83545.90 ± 1605.81^b^	13062.28 ± 939.76^b^	96608.18 ± 1863.55^a^
S8	2300.32 ± 90.12^b^	57.50 ± 1.55^c^	76301.61 ± 2050.62^c^	16448.152 ± 1413.53^a^	92749.76 ± 2475.07^ab^

Currency in the table is converted at a rate of 1 USD = 7.15 CNY. Different lowercase letters within the same column indicate significant differences at *P* < 0.05.

## 4 Discussion

### 4.1 Effects of soybean intercropping density on the photosynthetic characteristics of tobacco and soybean

This study demonstrated that soybean intercropping density significantly affected the photosynthetic characteristics of tobacco and simultaneously exerted a feedback influence on the photosynthetic performance of soybean itself. Overall, tobacco intercropped with four or six soybean hills exhibited significantly higher photosynthetic rates, chlorophyll fluorescence parameters, and pigment contents in both crops. In contrast, excessive density with eight soybean hills caused photosynthetic inhibition and restricted metabolism, indicating that an appropriate intercropping density can enhance light energy utilization efficiency by regulating canopy structure and resource allocation (Li et al., 2024b).

During the rosette stage of tobacco, the overall photosynthetic rate was low. However, in the treatments with four or six intercropped soybean hills, the net photosynthetic rate (Pn), stomatal conductance (Gs), and transpiration rate (Tr) were significantly higher than in monocropping, while the intercellular CO_2_ concentration (Ci) was lower, suggesting that photosynthetic limitation at this stage was mainly stomatal. Moderate intercropping improved inter-row ventilation and light conditions, promoting CO_2_ diffusion and stomatal regulation, thereby enhancing carbon assimilation efficiency (Fan et al., 2018; Dong et al., 2024). During the fast-growing stage, Pn increased markedly, with S4 and S6 treatments showing higher photosynthetic capacity, whereas the S8 treatment exhibited a sharp decline due to excessive canopy shading and insufficient light. At the maturity stage, Pn decreased overall, but moderate intercropping still maintained relatively high levels. In contrast, high-density intercropping showed decreased Gs but increased Ci, indicating that the limiting factor of photosynthesis shifted from stomatal to non-stomatal (Sakoda et al., 2021).

The photosynthetic responses of soybean followed a similar trend to those of tobacco. At the R2 and R4 stages, under the tobacco–soybean (four-hole) intercropping system, Pn, Gs, and Tr in soybean leaves were significantly higher than those under the eight-hole treatment, suggesting that moderate intercropping improved the light energy utilization and CO_2_ assimilation efficiency of soybean. Because the tobacco canopy exhibited relatively good light transmittance, moderate intercropping provided soybean plants with a more uniform light environment, helping to alleviate photoinhibition and delay leaf senescence (Wang et al., 2024). Under high-density intercropping, however, the lower soybean leaves were continuously exposed to low-light conditions, resulting in decreased photosynthetic rate and elevated intercellular CO_2_ concentration, indicating restricted carbon assimilation.

Chlorophyll fluorescence parameters reflect the activity of photosystem II (PSII) and energy conversion efficiency (Semer et al., 2019). In this study, tobacco intercropped with four and six soybean holes maintained higher maximum photochemical efficiency (Fv/Fm) and actual photochemical quantum yield (ΦPSII) at all growth stages, suggesting stronger PSII electron transport activity and more efficient light energy conversion (Li et al., 2009). Moderate intercropping can optimize the light distribution within the canopy, where proper shading reduces excessive light stress and the lower leaves receive more scattered light, thereby improving overall light-use efficiency (Chen et al., 2024). For soybean, the S4 treatment exhibited significantly higher Fv/Fm and ΦPSII at the R2, R4, and R6 stages compared with the eight-hole treatment, as the tobacco canopy served as a light buffer, mitigating photodamage and demonstrating a complementary and mutual light benefit effect (Zhang et al., 2021). Thus, a light environment characterized by both moderate shading and adequate transmittance can simultaneously enhance the photosynthetic efficiency of both main and intercrop species in intercropping systems (Brooker et al., 2015).

This study also found that moderate intercropping significantly increased the contents of chlorophyll a, chlorophyll b, and carotenoids in both tobacco and soybean leaves, especially in the four-hole intercropping treatment, where pigment levels remained high during the middle and late growth stages. The maintenance of pigment content not only enhances light-harvesting capacity but also helps delay chloroplast degradation, thereby slowing leaf senescence (Griffiths et al., 2014). Carotenoids play an important role in the antioxidant system by dissipating excess excitation energy through non-photochemical quenching (NPQ) and reducing the accumulation of reactive oxygen species (ROS), thus maintaining the structural stability of PSII (Latowski et al., 2011). In the eight-hole intercropping treatment, however, pigment contents in both tobacco and soybean decreased significantly, likely due to excessive shading and intensified nutrient competition, which accelerated chloroplast degradation and the aging of photosynthetic apparatus (Niinemets, 2023). In summary, a moderate intercropping density is essential for maintaining pigment homeostasis and sustaining efficient photosynthetic function in tobacco and soybean.

### 4.2 Effects of soybean intercropping density on carbon and nitrogen metabolism in tobacco

Rubisco, nitrate reductase (NR), and glutamine synthetase (GS) are key enzymes that connect photosynthetic carbon fixation with nitrogen assimilation. In the treatments with four and six intercropped soybean hills, the activities of these three enzymes were significantly higher than in monocropped tobacco, indicating that appropriate intercropping density enhanced the carbon and nitrogen metabolism of tobacco (Cong et al., 2015). The increase in Rubisco activity not only represents an enhancement of carboxylation capacity but also reflects a higher level of enzyme protein synthesis and activation (Iñiguez et al., 2021). This suggests that tobacco under moderate intercropping has a stronger capacity for CO_2_ fixation and energy supply, providing sufficient ATP and NADPH for subsequent nitrogen assimilation reactions (Kramer and Evans, 2011).

Meanwhile, the elevated activities of NR and GS indicate that nitrogen uptake, reduction, and assimilation processes were simultaneously enhanced, resulting in greater accumulation of nitrogen metabolites such as glutamine and glutamate in tobacco leaves. These compounds serve as essential substrates for amino acid and protein synthesis (Akhtar et al., 2024). Because tobacco leaves under moderate intercropping exhibit higher photosynthetic rates, the increased carbon skeletons produced can serve as substrates for NR and GS catalysis (Yoneyama and Suzuki, 2019). Moreover, the enhanced photosynthetic electron transport ensures sufficient NADPH supply from the light reactions, which strengthens NR reduction activity (Yamori et al., 2015). In addition to the stimulation of nitrogen assimilation by improved carbon metabolism, the availability and transformation efficiency of nitrogen itself were also enhanced by intercropping.

Soybean biological nitrogen fixation improves soil nitrogen availability. Through the secretion of organic acids, amino compounds, and flavonoids, soybean roots stimulate rhizosphere microbial activity, increasing the concentrations of ammonium nitrogen and soluble organic nitrogen in the tobacco root zone. These effects, in turn, promote the synthesis and activation of NR and GS (Lin et al., 2024; Molefe et al., 2023). Photosynthetic carbon metabolites also provide carbon skeletons for nitrogen assimilation, while enhanced nitrogen assimilation further facilitates the synthesis of key photosynthetic components such as Rubisco and photosystem proteins (Paul and Pellny, 2003). Under these positive feedback conditions, both photosynthetic productivity and nitrogen metabolism were simultaneously enhanced.

However, under the eight-hole intercropping treatment, excessive canopy density restricted light penetration and intensified belowground competition. Consequently, the synthesis and activation of Rubisco proteins declined, resulting in suppressed CO_2_ carboxylation rates (Zheng et al., 2022a). Moreover, the excessive root density of soybean increased soil oxygen consumption and led to ammonium accumulation, where high concentrations of NH_4_^+^ directly inhibited NR activity. In addition, hypoxic conditions in the rhizosphere impaired root metabolic function and induced rhizosphere acidification, further reducing NR and GS activities (Churchland and Grayston, 2014). These factors disrupted the coupling between carbon and nitrogen metabolism, causing metabolic imbalance. Therefore, a moderate intercropping density not only achieves complementarity in light energy and nutrient use at the canopy level but also maintains coordination between carbon–nitrogen flux and energy utilization at the cellular level. This coordination represents the fundamental physiological basis for the efficient operation of composite agricultural systems.

One limitation of this study is that only enzyme activities were analyzed, while gene expression and metabolite profiling were not included. Integrating transcriptomic and metabolomic analyses in future work would help clarify the molecular mechanisms underlying the coordination of carbon and nitrogen metabolism under different intercropping densities.

### 4.3 Effects of soybean intercropping density on the disease incidence

This study showed that in the tobacco–soybean intercropping system, moderate soybean intercropping effectively suppressed disease occurrence, whereas excessively high intercropping density produced the opposite effect. This indicates that there exists an optimal density range within the intercropping system that balances interspecific competition and mutualistic interactions.

Crop population density directly determines the transmission opportunities of pathogens. Moderate intercropping can reduce the infection probability by introducing non-host crops, thereby diluting the host density of the pathogen (Douma and Noordhoek, 2025). However, when the overall density becomes too high, the distance between plants decreases, effectively increasing the contact frequency among hosts and accelerating the spread of disease (Luo et al., 2021). Intercropping also influences disease development by altering the field microclimate. The inclusion of soybean modifies canopy ventilation and light penetration, which affects spore dispersal pathways and their survival in the air (Boudreau, 2013). Similar canopy-mediated effects have been reported in potato strip-planting systems, where intercropping significantly reduced the severity of late blight (Homulle et al., 2025). Conversely, excessive planting density leads to elevated canopy humidity and restricted airflow, providing favorable conditions for pathogen infection (Singh et al., 2023). This “density threshold effect” is consistent with findings by Wu et al. (2013) and Phillips (2005) on sheath blight in rice and late blight in potato.

Non-host crops can also indirectly suppress disease through rhizosphere microecological regulation. Isoflavonoid compounds secreted by soybean roots possess antifungal activity and can induce systemic resistance in tobacco (Yang et al., 2021; Sugiyama, 2019). In addition, root exudates from intercrop plants can reshape the rhizosphere microbial community, forming a disease-suppressive microecological network (Zhou et al., 2023b). Antagonistic microbial groups such as *Actinobacteria* and *Bacillus* species, enriched in the soybean rhizosphere, can inhibit soil-borne pathogens through competitive exclusion and secretion of antimicrobial metabolites (Lee et al., 2021; Li et al., 2024a). However, when soybean density is excessively high, intensified root competition may disrupt microbial community stability, potentially creating a favorable environment for pathogens (Yang et al., 2014).

In conclusion, moderate tobacco–soybean intercropping density suppresses tobacco black shank disease through a combination of host dilution, canopy regulation, and rhizosphere microecological optimization. This provides a theoretical basis for developing green and sustainable disease management strategies in tobacco cultivation systems.

### 4.4 Effects of soybean intercropping density on defense-related enzyme activities and signaling molecules in tobacco

The results of this study demonstrated that tobacco–soybean intercropping regulated the defense-related signaling molecules and resistance enzyme activities in tobacco, indicating that the system induced systemic resistance at the physiological and molecular levels. In the treatments with four or six intercropped soybean hills, the contents of salicylic acid (SA) and jasmonic acid (JA) increased significantly, accompanied by enhanced activities of phenylalanine ammonia-lyase (PAL) and antioxidant enzymes (POD and SOD). These findings suggest that intercropping enhanced the defensive potential of tobacco through the synergistic activation of the SA and JA signaling pathways (Qiu et al., 2014; Asghari and Hasanlooe, 2015).

SA and JA constitute two core signaling pathways of the plant immune system. SA primarily mediates systemic acquired resistance (SAR), whereas JA participates in induced systemic resistance (ISR) and defense against necrotrophic pathogens and herbivores (Ali et al., 2018; Heil and Bostock, 2002). Under moderate intercropping density, the simultaneous increase in SA and JA levels indicates that tobacco defense may not rely on a single pathway but rather on the coordinated activation of both, enabling simultaneous responses from SAR and ISR. Previous studies have shown that the SAR pathway induces the expression of pathogenesis-related (PR) proteins such as PR-1 and β-1,3-glucanase, while the ISR pathway upregulates transcription factors such as *WRKY* and *MYC2* via JA and ethylene signaling, thereby activating the antioxidant system (Darby et al., 2000; Javed and Gao, 2023). In this study, the significant enhancement of PAL, POD, and SOD activities was consistent with the changes in SA and JA levels, indicating that intercropping density may have strengthened systemic defense by activating these two signaling cascades. Root exudates from soybean, including flavonoids and phenolic acids, may serve as exogenous signaling molecules that activate the ISR pathway in tobacco roots (Mandal et al., 2010).

At the leaf level, SA accumulation enhances PAL pathway activity, promoting the phenylpropanoid metabolism toward the synthesis of lignin and phenolic compounds, thereby reinforcing structural resistance (Dixon et al., 2002). JA, on the other hand, enhances reactive oxygen signaling and induces the expression of antioxidant enzymes (SOD and POD), contributing to non-specific defense enhancement (Qiu et al., 2014). Therefore, under moderate intercropping density, positive crosstalk between SA and JA signaling likely results in synergistic enhancement of both systemic and local defense responses. In contrast, in the high-density treatment with eight intercropped soybean hills, the contents of SA and JA decreased significantly, and the activities of PAL and POD were suppressed, indicating that excessive density impaired the tobacco defense system.

In summary, the tobacco–soybean intercropping system activates the SA and JA signaling pathways, thereby inducing the coordinated expression of key enzymes in phenylpropanoid metabolism (such as PAL), PR protein-related defense genes, and antioxidant enzymes (POD and SOD). Together, these responses establish an integrated and efficient systemic defense network in tobacco.

### 4.5 Effects of soybean intercropping density on system yield and economic benefits

The results of this study showed that soybean intercropping density significantly affected the yield and economic benefits of the tobacco–soybean composite system. The treatment with four intercropped soybean hills per tobacco plant achieved the highest economic output per unit land area by increasing soybean income while maintaining stable tobacco quality. In this treatment, both tobacco and soybean contributed to the highest overall system benefit, as the increase in soybean yield complemented the stable economic return from tobacco.

The balance of chemical quality in flue-cured tobacco depends on the coordinated proportions of key components such as sugars, nitrogen, potassium, and chlorine. The treatment with four intercropped soybean hills significantly increased the total sugar and reducing sugar contents of tobacco leaves while maintaining low chlorine and appropriate nitrogen–alkaloid levels. This formed a “high-sugar, high-potassium, low-chlorine” compositional pattern, characteristic of high-quality flue-cured tobacco (Chen et al., 2021). The increase in sugar improves aroma and combustibility, whereas potassium accumulation buffers leaf acidity and enhances burning quality (Jiang et al., 2024; Banožić et al., 2020). These results are consistent with previous findings that intercropping can improve tobacco leaf quality (Gu et al., 2025), likely due to the enhancement of rhizosphere nutrient cycling and regulation of carbon–nitrogen metabolism by soybean roots (Nelson and Sadowsky, 2015; Liu et al., 2018). However, intercropping eight soybean hills per tobacco plant led to decreased sugar and potassium contents and increased chlorine content, likely because excessive planting density restricted photosynthesis and intensified nutrient competition, thereby reducing the accumulation and transformation efficiency of photosynthetic products in the leaves (Chen et al., 2019; Carrier et al., 2019).

The dynamic balance between competition and complementarity among crops is crucial for determining yield and economic efficiency in intercropping systems (Li et al., 2014). In this study, although monocropped tobacco had a higher individual yield value, the additional soybean income in the intercropping system significantly improved land use efficiency. Moderate intercropping achieved a “stable tobacco with enhanced soybean” effect, while excessive density reduced total system benefit due to inhibited tobacco growth and quality decline. Overall, intercropping tobacco with four soybean hills optimized both tobacco chemical composition and economic benefit, achieving the best balance between quality and efficiency. This configuration provides a theoretical basis for developing a green and complementary cropping system characterized by “tobacco supporting soybean and soybean enriching tobacco.”

## 5 Conclusion

This study demonstrated that an appropriate soybean intercropping density can markedly improve the photosynthetic performance of tobacco while simultaneously enhancing defense capacity and overall system productivity. Moderate intercropping significantly increased the net photosynthetic rate, chlorophyll content, and photosystem II efficiency of tobacco, and promoted carbon and nitrogen metabolism by elevating the activities of key enzymes such as Rubisco, nitrate reductase, and glutamine synthetase. Meanwhile, higher levels of salicylic acid (SA) and jasmonic acid (JA) collectively strengthened tobacco resistance and alleviated black shank disease. Among all treatments, intercropping four soybean hills per tobacco plant showed the best comprehensive performance. This configuration optimized canopy light distribution and resource utilization, improved photosynthetic efficiency and tobacco chemical quality, and formed a desirable “high-sugar, high-potassium, low-chlorine” composition. Consequently, it achieved optimal coordination among tobacco yield, quality, and system economic benefit. In summary, moderate soybean intercropping enhances photosynthetic productivity and physiological resilience in tobacco while improving the sustainability of the cropping system. Future studies should focus on multi-year and multi-regional validation of the optimized model and explore its underlying physiological and ecological mechanisms to support wider application.

## Data Availability

The original contributions presented in the study are included in the article/supplementary material. Further inquiries can be directed to the corresponding author.
